# A new test for canine intestinal mucosa permeability

**DOI:** 10.1186/1751-0147-57-S1-O16

**Published:** 2015-09-25

**Authors:** Ragnvi Hagman, Pernilla Rosberg, Anas Al-Saffar, Dominic Luc-Webb

**Affiliations:** 1 Department of Clinical Sciences, Swedish University of Agricultural Sciences, Uppsala, Sweden; 2 Department of Medical Sciences, Gastroenterology and Hepatology Unit, Uppsala University, Uppsala, Sweden

## Introduction

Increases in mucosal intestinal permeability may cause pathological leakage of bacterial products, inducing inflammation. Low-grade systemic inflammation has been reported in overweight humans. Probes used for permeability evaluation in humans have not yet been evaluated in dogs.

## Objective

To determine suitability of human permeability probes (riboflavin, lactulose, mannitol and sucralose) for canine obesity models.

## Methods

Fourteen healthy dogs were examined as baseline group. They were given all four probes *per os* using human doses corrected for weight and reported to be harmless for dogs. Urine samples were collected 2, 4 and 6 h after ingesting probes. Urinary excretion was quantified.

## Results

The least squares means of mannitol proportion (excreted/ingested ± SE) was 15.6 % (± 2.0), 11.0 % (± 2.1) and 5.5 % (± 2.1) for urine sampled 2, 4 and 6 h after ingestion (p<0.0001). Hence, the majority of mannitol absorption and secretion representing upper gastrointestinal leakage was completed by 4 h. Riboflavin followed a similar temporal profile, defining 4 h as a cutoff for upper vs lower gut permeability. Probes were well tolerated.

## Conclusions

Data obtained in the study indicates that probes used for humans can be used for intestinal permeability evaluation in dogs.

